# *DNAH11* compound heterozygous variants cause heterotaxy and congenital heart disease

**DOI:** 10.1371/journal.pone.0252786

**Published:** 2021-06-16

**Authors:** Hong Xia, Xiangjun Huang, Sheng Deng, Hongbo Xu, Yan Yang, Xin Liu, Lamei Yuan, Hao Deng

**Affiliations:** 1 Center for Experimental Medicine, the Third Xiangya Hospital, Central South University, Changsha, Hunan, China; 2 Department of Emergency, the Third Xiangya Hospital, Central South University, Changsha, Hunan, China; 3 Department of General Surgery, The First Affiliated Hospital of Hunan University of Chinese Medicine, Changsha, Hunan, China; 4 Department of Pharmacy, Xiangya Hospital, Central South University, Changsha, Hunan, China; 5 Department of Neurology, the Third Xiangya Hospital, Central South University, Changsha, Hunan, China; NIDCR/NIH, UNITED STATES

## Abstract

Heterotaxy (HTX), a condition characterized by internal organs not being arranged as expected relative to each other and to the left-right axis, is often accompanied with congenital heart disease (CHD). The purpose was to detect the pathogenic variants in a Chinese family with HTX and CHD. A non-consanguineous Han Chinese family with HTX and CHD, and 200 unrelated healthy subjects were enlisted. Exome sequencing and Sanger sequencing were applied to identify the genetic basis of the HTX family. Compound heterozygous variants, c.3426-1G>A and c.4306C>T (p.(Arg1436Trp)), in the dynein axonemal heavy chain 11 gene (*DNAH11*) were identified in the proband via exome sequencing and further confirmed by Sanger sequencing. Neither c.3426-1G>A nor c.4306C>T variant in the *DNAH11* gene was detected in 200 healthy controls. The *DNAH11* c.3426-1G>A variant was predicted as altering the acceptor splice site and most likely affecting splicing. The *DNAH11* c.4306C>T variant was predicted to be damaging, which may reduce the phenotype severity. The compound heterozygous variants, c.3426-1G>A and c.4306C>T, in the *DNAH11* gene might be the pathogenic alterations resulting in HTX and CHD in this family. These findings broaden the variant spectrum of the *DNAH11* gene and increase knowledge used in genetic counseling for the HTX family.

## Introduction

Human and other vertebrate visceral organs are asymmetric to the left-right (LR) axis. Normal organ placement relative to the LR axis is referred to as situs solitus (SS). Abnormal positioning is heterotaxy (HTX) or situs inversus (SI) [1]. A randomized placement of visceral organs relative to each other and LR axis is HTX. Visceral organ placement in a perfect mirror-image position along the LR axis is SI [2].

HTX is an extremely clinical heterogeneous disorder. HTX prevalence is about 1 in 10,000 newborns [3]. HTX, SI, and SS may present in different families or accidentally in the same family [1]. HTX is often connected with congenital heart disease (CHD) and is classified as either isolated or syndromic [4, 5]. At least 59 syndromes have been reported to associate either HTX or SI [5].

HTX is a genetic heterogeneous disorder with incomplete penetrance. It may be inherited in an autosomal recessive, autosomal dominant or X-linked fashion. It associates with genetic factors, environmental modifiers, and developmental randomness [4, 5]. As of the date of this writing (November 2020), nine loci (*HTX1-9*) and eight causative genes, including the Zic family member 3 gene (*ZIC3*), the cripto, FRL-1, cryptic family 1 gene (*CFC1*), the activin A receptor type 2B gene (*ACVR2B*), the nodal growth differentiation factor gene (*NODAL*), the cilia and flagella associated protein 53 gene (*CFAP53*), the matrix metallopeptidase 21 gene (*MMP21*), the polycystin 1 like 1, transient receptor potential channel interacting gene (*PKD1L1*) and the meiosis specific nuclear structural 1 gene (*MNS1*) have been reported as associating with isolated HTX [6–14].

The great clinical and genetic heterogeneity presents challenges to correctly identifying causative variants in individuals with HTX phenotype using Sanger sequencing. Exome sequencing, a cost-saving measure, has been used in the genetic investigation of HTX [12–14]. Our previous study has successfully identified pathogenic variants in two Chinese families with SI phenotype via exome sequencing [15, 16]. In this study, exome sequencing and Sanger sequencing detected the compound heterozygous variants, c.3426-1G>A and c.4306C>T (p.(Arg1436Trp)), in the dynein axonemal heavy chain 11 gene (*DNAH11*, NM_001277115.1) in a Han Chinese family with HTX and CHD.

## Materials and methods

### Subjects

**Fig 1:**
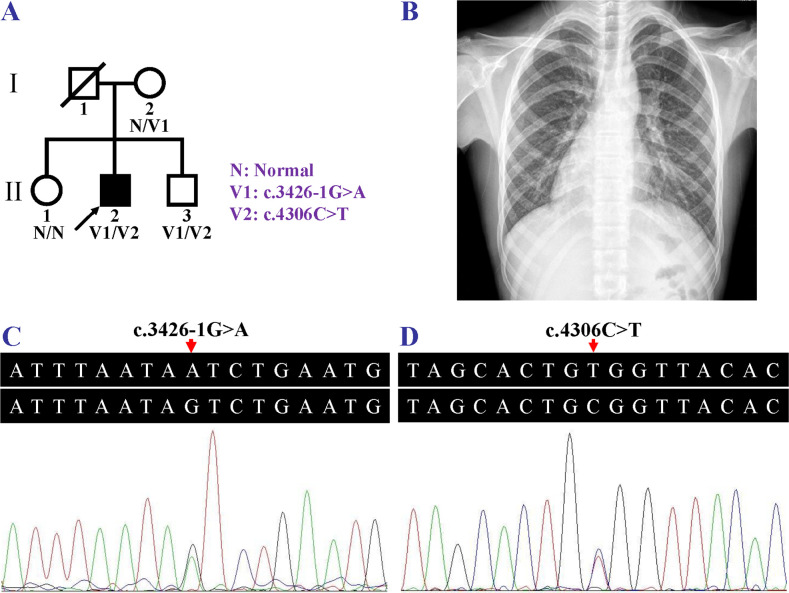
Pedigree of a Chinese heterotaxy family, chest X-ray of the heterotaxy patient and the *DNAH11* Sanger sequencing electropherograms. (A) Pedigree of a Chinese heterotaxy family. N, normal; V1, the *DNAH11* c.3426-1G>A variant; V2, the *DNAH11* c.4306C>T variant. The slash indicates deceased individual, the fully shaded symbol indicates the affected individual, and the arrow indicates the proband. (B) Chest X-ray of the family member (II:2) revealed dextrocardia. (C, D) The compound heterozygosity for the *DNAH11* variants, c.3426-1G>A and c.4306C>T, in the individual (II:2) with heterotaxy.

A two-generation, non-consanguineous Han family, from Yiyang, Hunan, China, with HTX and CHD was enrolled from May to July 2019. Four family members including the proband (II:2), his unaffected mother (I:2), and two unaffected siblings (II:1 and II:3) participated (Fig 1A). The proband (II:2) was diagnosed, at age five, as having cyanotic complex CHD due to dyspnea, cyanosis, poor activity endurance and cardiac murmurs. His first operation occurred at sixteen after he was admitted to hospital due to severe shortness of breath. He presented with cyanotic lips. The apical impulse was detected at the right fifth intercostal space. A systolic ejection murmur was heard in the right second intercostal space. Chest X-ray revealed dextrocardia (Fig 1B). Echocardiography showed complex CHD, including single ventricle, incomplete transposition of the great arteries, and pulmonary valve stenosis. No abdominal organ abnormalities were detected in the proband. Dyspnea, cyanosis, dextrocardia, cardiac murmurs, or cardiovascular malformations were not detected in his parents or other siblings. Two hundred unrelated subjects (100 males and 100 females, aged 16.5±5.5 years) who did not have dyspnea, cyanosis, dextrocardia, or cardiovascular anomalies were recruited as healthy controls from May to October 2019. The study was approved by the Institutional Review Board of the Third Xiangya Hospital, Central South University (Changsha, China) in accordance with the Declaration of Helsinki. The study was conducted from May to December 2019. Written informed consent forms were obtained, and venous blood samples were taken from all participants. The authors had no access to information that could identify individual participants during or after data collection.

### Exome sequencing

Genomic DNA was obtained from blood samples via a saturated phenol-chloroform extraction method [17]. A microgram of genomic DNA from the proband (II:2) was exome sequenced at BGI-Shenzhen, China. The library with targeted exome was captured by the Agilent SureSelect Human All Exon V6 and sequenced using a BGISEQ-500 platform. Average sequencing depth was 267.63×. Debased reads were filtered and the clean reads were mapped to the human reference genome (UCSC database version hg19, http://genome.ucsc.edu/) using a Burrows-Wheeler Aligner tool (BWA, version 0.7.15). Duplicate reads were marked by Picard-tools (version 2.5.0). Single nucleotide polymorphisms (SNPs) and insertions/deletions (Indels) were detected by a HaplotypeCaller of Genome Analysis Toolkit (GATK, version 3.3.0). Variant annotation and prediction were performed by SnpEff tool (https://pcingola.github.io/SnpEff/). Variants with minor allele frequency ≥1% in the following databases were eliminated: the SNP database version 141 (dbSNP141), the 1000 Genomes Project (1000 genomes release phase 3), and the NHLBI Exome Sequencing Project (ESP) 6500 [18]. The functional impacts of nonsynonymous SNPs or Indels were predicted by the Sorting Intolerant from Tolerant (SIFT) and the Protein Variation Effect Analyzer (PROVEAN, version 1.1.3, http://provean.jcvi.org/index.php). The effects of splice site variants on splicing were predicted by the Human Splicing Finder (HSF, version 3.1, http://www.umd.be/HSF3/).

### Sanger sequencing, variant analysis and protein structure modeling

Candidate variants were validated by Sanger sequencing. A co-segregation analysis between possible causative variants and this family’s HTX phenotype was performed [19]. Paired primer sequences for the candidate variants (c.3426-1G>A and c.4306C>T) in the *DNAH11* gene were synthesized respectively as follows: 5′-TGTTGCCAGTTTCATGATAGAGA-3′ and 5′-TACAGCCAGAAGATGCACCA-3′; 5′-TTCACCAGCCTTTAGGCAAA-3′ and 5′-TCTCAGTCCCCAGCTCTTTC-3′. The recorded frequencies of the candidate variants in the Genome Aggregation Database (gnomAD, version 3.0, http://gnomad.broadinstitute.org) were further checked, and pathogenic variant databases, including the Leiden Open Variation Database (LOVD, v.3.0, https://www.lovd.nl/3.0/home), the Human Gene Mutation Database (HGMD, http://www.hgmd.cf.ac.uk/ac/index.php), and ClinVar (https://www.ncbi.nlm.nih.gov/clinvar/) were referred to interpreting variants. The American College of Medical Genetics and Genomics (ACMG) sequence variant interpretation guidelines, and recommendations for interpreting the loss of function pathogenic criterion (PVS1) created by ClinGen Sequence Variant Interpretation Working Group, were used to categorize the identified variants [20, 21].

The protein structures of wild-type and variant-type were predicted via the online SWISS-MODEL tool (http://www.swissmodel.expasy.org), and the visualized structures were constructed using the PyMOL software (version 1.7, Schrödinger, LLC, Portland, U.S.A.).

## Results

Exome sequencing generated 240,827,260 clean reads. Approximately 99.94% of the clean reads were mapped to the human reference genome. A total of 105,788 SNPs and 18,688 Indels were detected. After a series of optimization screening measures, two variants, c.3426-1G>A and c.4306C>T in the *DNAH11* gene, were discovered. Other possible disease-causing alterations in the pathogenic genes known for HTX, SI or CHD were eliminated.

The compound heterozygous variants, a splicing variant, c.3426-1G>A, and a missense variant, c.4306C>T (p.(Arg1436Trp)), in the *DNAH11* gene, were further confirmed in the HTX patient (II:2) via Sanger sequencing (Fig 1C and 1D). The heterozygous c.3426-1G>A variant in the *DNAH11* gene was detected in his mother (I:2). Neither c.3426-1G>A nor c.4306C>T variant in the *DNAH11* gene was discovered in his elder sister (II:1). The compound heterozygous variants, c.3426-1G>A and c.4306C>T, in the *DNAH11* gene were also identified in his brother (II:3) who had no HTX or SI phenotype. This was in accord with prior reports that SI and HTX may occur in about half of PCD patients, approximately 59.1% of individuals or 42.9% of affected siblings having the *DNAH11* biallelic variants [22, 23]. His father’s genotyping was indefinable as he (I:1) passed away. Due to the presence of the *DNAH11* c.4306C>T variant in two brothers (II:2 and II:3) and the absence in the mother (I:2), the variant might be not *de novo*, but inherited from his father (I:1), further supporting that the disorder was inherited in an autosomal recessive manner in this family. Neither *DNAH11* c.3426-1G>A nor c.4306C>T variant was detected in the 200 healthy controls.

**Fig 2:**
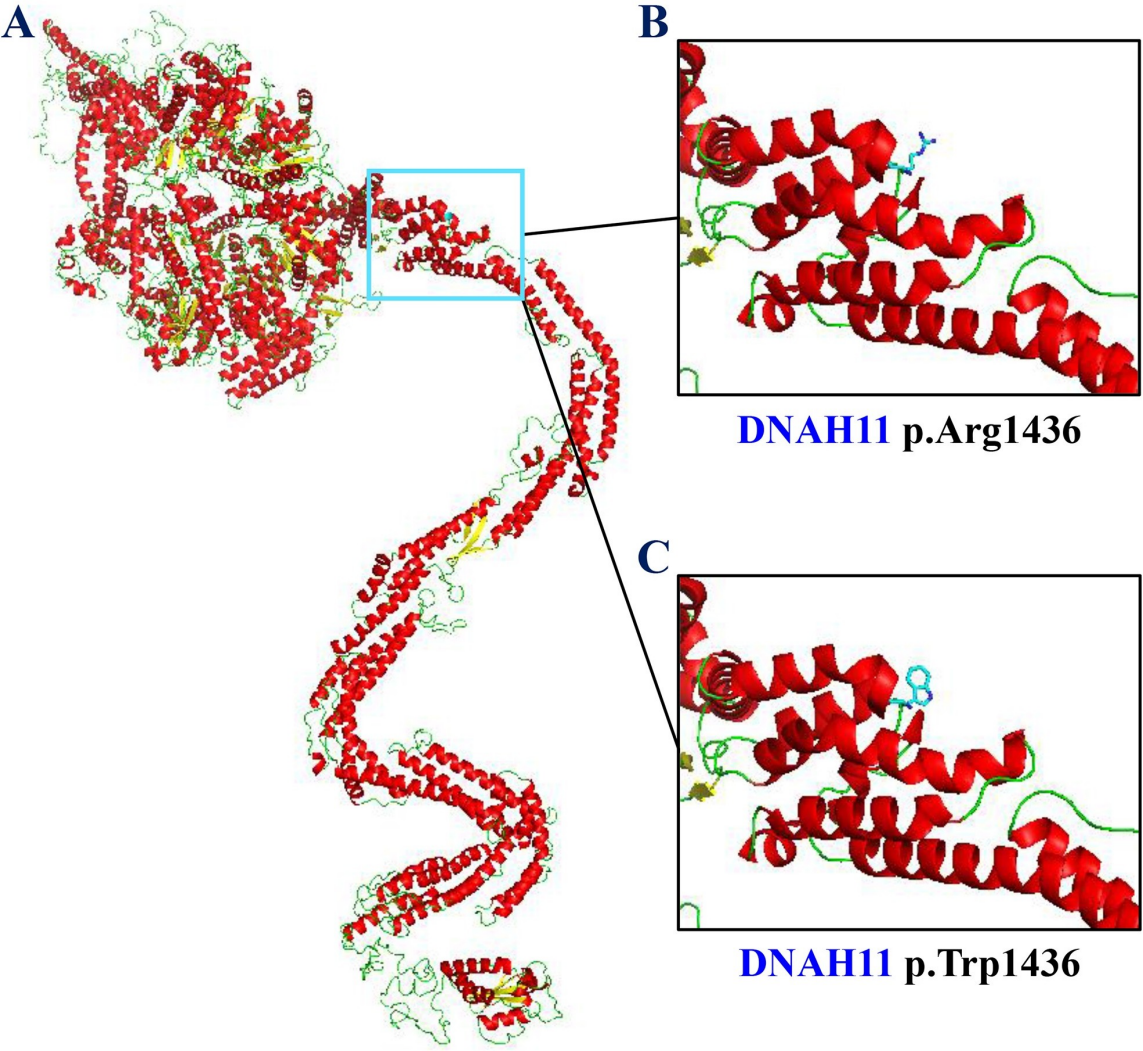
Structural modeling displayed conformational changes of the DNAH11 p.(Arg1436Trp) variant. (A) The cartoon representation of the DNAH11 protein. (B) The stick model of the arginine at residue 1436 (p.Arg1436). (C) The stick model of the mutant tryptophan at residue 1436 (p.Trp1436).

The HSF tool predicted that the *DNAH11* c.3426-1G>A variant alters acceptor splice site and most probably affects splicing. The *DNAH11* c.4306C>T variant was predicted to result in substituting tryptophan for arginine at codon 1436 (p.(Arg1436Trp)) and it would be damaging with a prediction score of 0.001 by SIFT, and be deleterious with a score of -3.10 by PROVEAN. The allele frequencies in the gnomAD were 1.31×10^−5^ for c.3426-1G>A and 3.09×10^−4^ for c.4306C>T. The two variants were not recorded in LOVD, but deposited in HGMD, responsible for ciliary dyskinesia and bronchiectasis, respectively. The *DNAH11* c.3426-1G>A variant was interpreted as pathogenic in the ClinVar database, while the c.4306C>T variant had conflicting interpretations of pathogenicity (uncertain significance or benign). According to guidelines for the sequence variant interpretation, the variants, c.3426-1G>A and c.4306C>T, were classified as “pathogenic” (PVS1+PM2+PP5) and “uncertain significance” (PM2), respectively. Conformational changes after the arginine at residue 1436 (p.Arg1436) altered to tryptophan (p.Trp1436) were displayed by structural modeling (Fig 2).

## Discussion

HTX occurs in approximately 6.3%-12.1% of patients having primary ciliary dyskinesia (PCD) [24, 25]. In 2002, a homozygous c.8533C>T (p.(Arg2845*)) variant and a heterozygous c.8990G>A (p.(Arg2997Gln)) variant in the *DNAH11* gene were firstly detected in two patients with SI and most likely PCD, respectively [26]. Subsequently compound heterozygous variants, c.12363C>G (p.(Tyr4121*)) and c.13531_*36del (p.(Ala4511_Ala4516delinsGln)), in the *DNAH11* gene were identified in a family with PCD, including a patient with SI [27]. Biallelic variants in the *DNAH11* gene may be responsible for about 22% of PCD patients with normal ciliary ultrastructure. It was reported that SI occurred in 54.5% of PCD patients, HTX in about 4.5% of PCD patients, and SS in about 41% of PCD patients with *DNAH11* biallelic variants [22].

The *DNAH11* gene locates in 7p15.3, contains 82 exons, and encodes a ciliary outer dynein arm with 4516 amino acids. The protein contains an N-terminal tail domain, six ATPase associated diverse cellular activities (AAA) domains, four P-loops, a helix-1-microtubule-binding-helix-2 domain, and a C-domain [26].

The embryonic expression of *Dnah11*, a mouse homolog of human *DNAH11*, also called “left-right dynein” (*lrd*), is detected primarily in non-ciliated cells and highly restricted in the node at mouse embryonic day 7.5. It also expresses in many ciliated cells in newborn and adult mice [28]. Dnah11-containing monocilia in the node generate a leftward nodal flow which may determine the normal left-right patterns in mouse embryos [29, 30].

The *iv*/*iv* mice, resulting from the homozygous missense p.Glu2271Lys variant in the *Dnah11* gene, presented with an abnormal nodal flow, randomized expression of lateralized genes, a high incidence of cardiovascular anomaly, and randomized left-right development. Half of them exhibited either HTX or SI, and half exhibited SS [28, 30–34]. Phenotypes indicative of human PCD, including rhinitis, sinusitis, otitis media, static tracheal cilia, and sperm motility defects, were also discovered in the *iv/iv* mice [34].

Compound heterozygous *DNAH11* variants, responsible for about 14.3% of patients with CHD and HTX, may be a frequent genetic basis of familial CHD and HTX syndrome [35]. The reported compound heterozygous variants in the *DNAH11* gene and their various phenotypes were summarized in Table 1 [22, 23, 27, 34–43].

**Table 1:** Clinical phenotypes of individuals with *DNAH11* compound heterozygous variants.

Patients	Gender	Ethnicity	Sequence variants^#^	PCD phenotypes	Situs	CHD	Ciliary motion	References
-	F	Chinese	c.73G>A, p.(Ala25Thr)	BR, rhinosinusitis	SI	-	KS	[36]
			c.5702A>C, p.(Glu1901Ala)					
OP41-II:1	M	Caucasian	c.350A>T, p.(Glu117Val)	NRD, sinusitis, OM	SI	-	KS (H)	[22]
			c.7148T>C, p.(Leu2383Pro)					
#5062	F	Chinese	c.727A>G, p.(Ile243Val)	na	HTX	+	CD (I+R)	[35]
			c.10829A>T, p.(Asp3610Val)					
#5031	M	Chinese	c.846G>C, p.(Met282Ile)	na	HTX	+	CD (I+R+D)	[35]
P44	M		c.2406G>A, p.(Trp802*)	Rhinosinusitis	HTX	+	KS (R)	[37]
A	F	Italian	c.883-1G>A	A: BR, rhinitis, rhinosinusitis, otitis	SI	-	KS (I+H)	[38]
B	M		c.4130G>A, p.(Trp1377*)		SI			
				B: Recurrent pneumonia, BR				
B212	na	Chinese	c.1300T>C, p.(Phe434Leu)	BR	na	na	na	[39]
			c.6983C>T, p.(Pro2328Leu)					
#5065	M	Chinese	c.1339G>A, p.(Gly447Arg)	na	HTX	+	CD (I+R)	[35]
			c. 3470T>G, p.(Leu1157Arg)					
Patient 1	M	Polish	c.1648del, p.(Arg550Glyfs*16)	na	HTX	+	na	[40]
Patient 2	na		c.2772G>A, p.(Met924Ile)					
			c.11662C>T, p.(Arg3888Cys)					
PCD761	F	Caucasian	c.2275-1G>C, p.Tyr759_Glu889del	NRD, BR, sinusitis, OM	SI	-	KS (H)	[22]
			c.13213del, p.(Arg4405Alafs*2)					
Family 1–1	F	Finnish	c.2341G>A, p.(Glu781Lys)	Rhinosinusitis, OM	SI	-	KS (Static) PCD (D)	[41]
Family 1–2	M		c.7645+5G>A	Rhinosinusitis	SS			
B082	na	Chinese	c.2419G>C, p.(Asp807His)	BR	na	na	na	[39]
			c.2542G>A, p.(Val848Met)					
B012	na	Chinese	c.2419G>C, p.(Asp807His)	BR	na	na	na	[39]
			c.12258C>A, p.(Tyr4086*)					
P28	M	Chinese	c.2485C>T, p.(Arg829Cys)	Rhinosinusitis, OM	SS	-	PCD (R)	[37]
			c.5608C>T, p.(Pro1870Ser)					
B036	na	Chinese	c.2542G>A, p.(Val848Met)	BR	na	na	na	[39]
			c.9260A>G, p.(Lys3087Arg)					
B170	na	Chinese	c.2542G>A, p.(Val848Met)	BR	na	na	na	[39]
			c.11624A>G, p.(Lys3875Arg)					
B156	na	Chinese	c.2542G>A, p.(Val848Met)	BR	na	na	na	[39]
			c.12071C>T, p.(Ser4024Phe)					
11174	M	Italian	c.2753G>T, p.(Gly918Val)	Bronchitis, rhinitis, sinusitis, otitis	SS	-	PCD (I)	[23]
			c.12796_12801delinsATA, p.Phe4266_Asn4267delinsIle					
#4	F	English	c.2832dup, p.(Gln945Serfs*10)	Recurrent chest infection, rhinitis	SS	-	PCD	[42]
			c.13240dup, p.(Thr4414Asnfs*34)					
B142	na	Chinese	c.2912A>G, p.(Asp971Gly)	BR	na	na	na	[39]
			c.11396T>C, p.(Ile3799Thr)					
P25	M	Chinese	c.3020T>G, p.(Leu1007*)	Rhinosinusitis, OM	SI	-	KS (N)	[37]
			c.3470T>G, p.(Leu1157Arg)					
#6	F	English	c.3220G>T, p.(Glu1074*)	Recurrent chest infection	SI	-	KS (Static)	[42]
			c.13069C>T, p.(Arg4357*)					
II:2	M	Chinese	c.3426-1G>A	-	HTX	+	na	This study
			c.4306C>T, p.(Arg1436Trp)					
P45	F	Chinese	c.3470T>G, p.(Leu1157Arg)	Rhinosinusitis, OM	SI	-	KS (R)	[37]
			c.6727C>G, p.(Arg2243Gly)					
#7	M	English	c.3544C>T, p.(Arg1182*)	Recurrent chest infection, rhinitis	SS	-	PCD	[42]
			c.8798-5G>A					
#1	M	English	c.3727G>T, p.(Glu1243*)	Recurrent chest infection, rhinitis	SS	-	PCD (Static)	[42]
			c.13531_13532insTTCAGGCTGAAGA, p.(Ala4511Valfs*13)					
PCD1077	F	Caucasian	c.3901G>T, p.(Glu1301*)	NRD, sinusitis, OM	SI	-	KS	[22]
			c.11804C>T, p.(Pro3935Leu)					
OP406-II:1	M	Caucasian	c.4254+5G>T	na	SI		KS (H)	[22]
OP406-II:2	F		c.4726-1G>A	NRD, sinusitis	SS		PCD (H)	
B099	na	Chinese	c.4306C>T, p.(Arg1436Trp)	BR	na	na	na	[39]
			c.6118C>T, p.(Arg2040Cys)					
#2	F	English	c.4395_4398del, p.(Ser1465Argfs*6)	Recurrent chest infection, rhinitis	SS	-	PCD (Static)	[42]
			c.7642C>T, p.(Gln2548*)					
P32	M	Chinese	c.4457T>A, p.(Leu1486Gln)	NRD, rhinosinusitis, OM	SS	-	PCD (N)	[37]
			c.10006G>T, p.(Ala3336Ser)					
9003	F	American	c.4505A>C, p.(Gln1502Pro)	NRD, recurrent pneumonia, BR, sinusitis, OM	HTX	+	CD (H)	[43]
			c.9376G>A, p.(Glu3126Lys)					
PCD106	M	Caucasian	c.4516_4517del, p.(Leu1506Serfs*11)	Sinusitis, OM	SS	-	PCD	[22]
							KS (H)	
PCD108	M			NRD, sinusitis, OM	SI			
			c.7266+1G>A					
B185	na	Chinese	c.4898C>A, p.(Ser1633Tyr)	BR	na	na	na	[39]
			c.9260A>G, p.(Lys3087Arg)					
11228	M	Italian	c.4922C>G, p.(Ser1641*)	Bronchitis, rhinitis	SI	-	KS	[23]
			c.9304G>A, p.(Gly3102Ser)					
#5707	M	Chinese	c.5473dup, p.(Gln1825Profs*23)	na	HTX	+	CD (I+R)	[35]
			c.8275T>C, p.(Phe2759Leu)					
			c.13183C>T, p.(Arg4395*)					
#3	F	English	c.5506C>T, p.(Arg1836*)	Recurrent chest infection	SI	-	KS (Static)	[42]
			c.5636T>A, p.(Leu1879*)					
PCD565	M	Caucasian	c.5778+1G>A	NRD, BR, sinusitis, OM	SI	-	KS (H)	[22]
			c.13061T>A, p.(Leu4354His)					
PCD812	M	Caucasian	c.5815G>A, p.(Gly1939Arg)	NRD, sinusitis, OM	SI	-	KS	[22]
			c.13373C>T, p.(Pro4458Leu)					
PCD157	F	Caucasian	c.6244C>T, p.(Arg2082*)	NRD, BR, sinusitis, OM	SI	-	KS (H)	[22]
			c.11929G>T, p.(Glu3977*)					
#5130	F	Chinese	c.6785T>C, p.(Ile2262Thr)	na	HTX	+	CD (R+D)	[35]
			c.11398G>C, p.(Asp3800His)					
B178	na	Chinese	c.6905A>C, p.(His2302Pro)	BR	na	na	na	[39]
			c.13112C>T, p.(Pro4371Leu)					
B073	na	Chinese	c.7292G>T, p.(Ser2431Ile)	BR	na	na	na	[39]
			c.9017C>T, p.(Thr3006Met)					
P17	M	Chinese	c.7292G>T, p.(Ser2431Ile)	NRD, rhinosinusitis, OM	SI	-	KS (R)	[37]
P30	M				SS	+	PCD (R)	
			c.7364A>C, p.(Asp2455Ala)	NRD				
			c.9017C>T, p.(Thr3006Met)					
			c.13373C>T, p.(Pro4458Leu)					
#730	na	Caucasian	c.7772C>T, p.(Pro2591Leu)	NRD, rhinitis, sinusitis, OM	SS	-	PCD (erratic)	[34]
			c.8698C>T, p.(Arg2900*)					
OP98-II:1	M	Caucasian	c.7914G>C, p.Trp2604*	BR, sinusitis, OM	SI	-	KS (H)	[22]
							PCD (H)	
OP98-II:2	M		c.13330_13333dup, p.(Ile4445Asnfs*4)		SS			
C	M	Italian	c.8114A>G, p.(His2705Arg)	NRD, recurrent pneumonia, sinusitis, otitis	SI	+	KS (I+H)	[38]
			c.10264G>A, p.(Gly3422Arg)					
B167	na	Chinese	c.9260A>G, p.(Lys3087Arg)	BR	na	na	na	[39]
			c.11647C>T, p.(Leu3883Phe)					
B163	na	Chinese	c.9260A>G, p.(Lys3087Arg)	BR	na	na	na	[39]
			c.13175C>T, p.(Thr4392Met)					
P23	F	Chinese	c.9539T>A, p.(Leu3180*)	Rhinosinusitis, OM	SI	-	KS (R)	[37]
			c.9706C>T, p.(Arg3236*)					
#5045	M	Chinese	c.10379C>A, p.(Thr3460Lys)	na	HTX	+	CD (R+D)	[35]
			c.13273G>A, p.(Gly4425Ser)					
PCD1126	F	Asian	c.12064G>C, p.(Ala4022Pro)	BR, sinusitis	SS	-	PCD (H)	[22]
			c.13500_13504dup, p.(Thr4502Argfs*15)					
II-2	M	German	c.12363C>G, p.Tyr4121*	Recurrent pneumonia, bronchitis, sinusitis, BR (II-4, 6, 11), OM (II-3, 4, 9)	SI	-	KS	[27]
							PCD	
							PCD	
II-3, 4	F		c.13531_*36del, p.Ala4511_Ala4516delinsGln		SS			
II-6, 9, 11	M				SS			
OP235-II:1	F	Caucasian	c.12697C>T, p.(Gln4233*)	BR, sinusitis, OM	SS	-	PCD (H)	[22]
							KS (H)	
OP235-II:2	F		c.12980T>C, p.(Leu4327Ser)	NRD, BR, sinusitis, OM	SI			
PCD918	F	Asian	c.13065_13067del, p.(Leu4356del)	BR, NRD, sinusitis, OM	SS	-	PCD	[22]
							KS (H)	
PCD919	M				HTX			
			c.13075C>T, p.(Arg4359*)					

^#^ Description of the sequence variants is recalibrated in accordance with the Human Genome Variation Society nomenclature for variants (http://varnomen.hgvs.org/) using the reference sequence (NM_001277115.1).

BR, bronchiectasis; CD, ciliary dysfunction; CHD, congenital heart disease; D, discordance; F, female; H, hyperkinetic; HTX, heterotaxy; I, immotile; KS, Kartagener syndrome; M, male; N, nearly normal; na, not available; NRD, neonatal respiratory distress; OM, otitis media; PCD, primary ciliary dyskinesia; R, restricted; SI, situs inversus; SS, situs solitus; +, present; -, not present.

In this study, the compound heterozygous variants, c.3426-1G>A and c.4306C>T (p.(Arg1436Trp)), in the *DNAH11* gene were identified in the HTX patient and his unaffected brother. Different clinical phenotypes (HTX, SI or SS) of the siblings with the *DNAH11* gene biallelic variants have been reported in some families [22, 40]. HTX and SI occurring in about 59% of individuals with the *DNAH11* biallelic variants may be connected with clinical heterogeneity of LR asymmetry disorders with incomplete penetration, or additional genetic variants in the family member II:3 serving a suppressor in the *DNAH11* pathway. It might also be associated with environmental modifiers and randomized left-right development [1, 22].

The *DNAH11* c.3426-1G>A variant in the homozygous state was previously identified in a fetus with structural abnormalities, including single atrium and ventricle, pulmonary stenosis, and right isomerism [44]. The compound heterozygous *DNAH11* missense variants, c.4306C>T (p.(Arg1436Trp)) and c.6118C>T (p.(Arg2040Cys)), were reported in a patient with bronchiectasis, a clinical manifestation of PCD [39]. Neither variants were detected in the enrolled 200 healthy controls. Allele frequency of either variant in the total population was very low. The *DNAH11* c.3426-1G>A variant was predicted to alter the acceptor splice site, and the c.4306C>T variant was predicted to be damaging. Together with the pathogenicity evidence, the c.3426-1G>A and c.4306C>T variants in the *DNAH11* gene, as a novel combination, could be the pathogenic variants for HTX and CHD in this family, and the missense variant may reduce the phenotype severity.

*DNAH11* biallelic variants were detected in patients with ciliary dysfunction, HTX, SI and CHD phenotypes [23, 40]. The patient refused to permit either the nasal curettage or the bronchial mucosal biopsy to perform an analysis on the ciliary ultrastructure and the waveform. No obvious chronic airway infection symptoms or signs, such as recurrent rhinitis, sinusitis, otitis media, bronchitis, frequent pneumonias, or bronchiectasis were observed in the two family members having the *DNAH11* compound heterozygous variants. However, the very minor or preclinical phenotypes of PCD, such as rhinitis and bronchitis, might be overlooked and a diagnosis of mild PCD may be not excluded. In this study, there is a limitation that the father’s genotyping is unavailable due to his death. He died from an accidental injury, without complaining of PCD-related rhinitis, sinusitis, otitis media, bronchitis, bronchiectasis, or cardiovascular anomaly.

In conclusion, the compound heterozygous variants, c.3426-1G>A and c.4306C>T, in the *DNAH11* gene might be the pathogenic alterations in this family with HTX and CHD. Our findings broaden the variant spectrum of the *DNAH11* gene and provide additional information usable in genetic counseling for this family.
