# Correction: *DNAH11* compound heterozygous variants cause heterotaxy and congenital heart disease

**DOI:** 10.1371/journal.pone.0315965

**Published:** 2024-12-12

**Authors:** Hong Xia, Xiangjun Huang, Sheng Deng, Hongbo Xu, Yan Yang, Xin Liu, Lamei Yuan, Hao Deng

There is an error in the third sentence of the first paragraph of the Discussion. The correct sentence is: Subsequently compound heterozygous variants, c.12363C>G (p. Tyr4121*) and c.13531_*36del (p.Ala4511_Ala4516delinsGln), in the *DNAH11* gene were identified in a family with PCD, including a patient with SI.

Additionally, in Table 1, the columns Ethnicity, Sequence variants # and PCD phenotypes are incorrectly formatted. Please see the correct version of Table 1 here.

**Table 1:** Clinical phenotypes of individuals with DNAH11 compound heterozygous variants.

Patients	Gender	Ethnicity	Sequence variants^#^	PCD phenotypes	Situs	CHD	Ciliary motion	References
-	F	Chinese	c.73G>A, p.(Ala25Thr)	BR, rhinosinusitis	SI	-	KS	[36]
			c.5702A>C, p.(Glu1901Ala)					
OP41-II:1	M	Caucasian	c.350A>T, p.(Glu117Val)	NRD, sinusitis, OM	SI	-	KS (H)	[22]
			c.7148T>C, p.(Leu2383Pro)					
#5062	F	Chinese	c.727A>G, p.(Ile243Val)	na	HTX	+	CD (I+R)	[35]
			c.10829A>T, p.(Asp3610Val)					
#5031	M	Chinese	c.846G>C, p.(Met282Ile)	na	HTX	+	CD (I+R+D)	[35]
P44	M		c.2406G>A, p.(Trp802*)	Rhinosinusitis	HTX	+	KS (R)	[37]
A	F	Italian	c.883-1G>A	BR, rhinitis, rhinosinusitis, otitis	SI	-	KS (I+H)	[38]
B	M		c.4130G>A, p.(Trp1377*)	Recurrent pneumonia, BR				
B212	na	Chinese	c.1300T>C, p.(Phe434Leu)	BR	na	na	na	[39]
			c.6983C>T, p.(Pro2328Leu)					
#5065	M	Chinese	c.1339G>A, p.(Gly447Arg)	na	HTX	+	CD (I+R)	[35]
			c. 3470T>G, p.(Leu1157Arg)					
Patient 1	M	Polish	c.1648del, p.(Arg550Glyfs*16)	na	HTX	+	na	[40]
Patient 2	na		c.2772G>A, p.(Met924Ile)					
			c.11662C>T, p.(Arg3888Cys)					
PCD761	F	Caucasian	c.2275-1G>C, p.Tyr759_Glu889del	NRD, BR, sinusitis, OM	SI	-	KS (H)	[22]
			c.13213del, p.(Arg4405Alafs*2)					
Family 1–1	F	Finnish	c.2341G>A, p.(Glu781Lys)	Rhinosinusitis, OM	SI	-	KS (Static)	[41]
Family 1–2	M		c.7645+5G>A	Rhinosinusitis	SS		PCD (D)	
B082	na	Chinese	c.2419G>C, p.(Asp807His)	BR	na	na	na	[39]
			c.2542G>A, p.(Val848Met)					
B012	na	Chinese	c.2419G>C, p.(Asp807His)	BR	na	na	na	[39]
			c.12258C>A, p.(Tyr4086*)					
P28	M	Chinese	c.2485C>T, p.(Arg829Cys)	Rhinosinusitis, OM	SS	-	PCD (R)	[37]
			c.5608C>T, p.(Pro1870Ser)					
B036	na	Chinese	c.2542G>A, p.(Val848Met)	BR	na	na	na	[39]
			c.9260A>G, p.(Lys3087Arg)					
B170	na	Chinese	c.2542G>A, p.(Val848Met)	BR	na	na	na	[39]
			c.11624A>G, p.(Lys3875Arg)					
B156	na	Chinese	c.2542G>A, p.(Val848Met)	BR	na	na	na	[39]
			c.12071C>T, p.(Ser4024Phe)					
11174	M	Italian	c.2753G>T, p.(Gly918Val)	Bronchitis, rhinitis, sinusitis, otitis	SS	-	PCD (I)	[23]
			c.12796_12801delinsATA, p.Phe4266_Asn4267delinsIle					
#4	F	English	c.2832dup, p.(Gln945Serfs*10)	Recurrent chest infection, rhinitis	SS	-	PCD	[42]
			c.13240dup, p.(Thr4414Asnfs*34)					
B142	na	Chinese	c.2912A>G, p.(Asp971Gly)	BR	na	na	na	[39]
			c.11396T>C, p.(Ile3799Thr)					
P25	M	Chinese	c.3020T>G, p.(Leu1007*)	Rhinosinusitis, OM	SI	-	KS (N)	[37]
			c.3470T>G, p.(Leu1157Arg)					
#6	F	English	c.3220G>T, p.(Glu1074*)	Recurrent chest infection	SI	-	KS (Static)	[42]
			c.13069C>T, p.(Arg4357*)					
II:2	M	Chinese	c.3426-1G>A	-	HTX	+	na	This study
			c.4306C>T, p.(Arg1436Trp)					
P45	F	Chinese	c.3470T>G, p.(Leu1157Arg)	Rhinosinusitis, OM	SI	-	KS (R)	[37]
			c.6727C>G, p.(Arg2243Gly)					
#7	M	English	c.3544C>T, p.(Arg1182*)	Recurrent chest infection, rhinitis	SS	-	PCD	[42]
			c.8798-5G>A					
#1	M	English	c.3727G>T, p.(Glu1243*)	Recurrent chest infection, rhinitis	SS	-	PCD (Static)	[42]
			c.13531_13532insTTCAGGCTGAAGA, p.(Ala4511Valfs*13)					
PCD1077	F	Caucasian	c.3901G>T, p.(Glu1301*)	NRD, sinusitis, OM	SI	-	KS	[22]
			c.11804C>T, p.(Pro3935Leu)					
OP406-II:1	M	Caucasian	c.4254+5G>T	na	SI	-	KS (H)	[22]
OP406-II:2	F		c.4726-1G>A	NRD, sinusitis	SS		PCD (H)	
B099	na	Chinese	c.4306C>T, p.(Arg1436Trp)	BR	na	na	na	[39]
			c.6118C>T, p.(Arg2040Cys)					
#2	F	English	c.4395_4398del, p.(Ser1465Argfs*6)	Recurrent chest infection, rhinitis	SS	-	PCD (Static)	[42]
			c.7642C>T, p.(Gln2548*)					
P32	M	Chinese	c.4457T>A, p.(Leu1486Gln)	NRD, rhinosinusitis, OM	SS	-	PCD (N)	[37]
			c.10006G>T, p.(Ala3336Ser)					
9003	F	American	c.4505A>C, p.(Gln1502Pro)	NRD, recurrent pneumonia, BR, sinusitis, OM	HTX	+	CD (H)	[43]
			c.9376G>A, p.(Glu3126Lys)					
PCD106	M	Caucasian	c.4516_4517del, p.(Leu1506Serfs*11)	Sinusitis, OM	SS	-	PCD	[22]
PCD108	M		c.7266+1G>A	NRD, sinusitis, OM	SI		KS (H)	
B185	na	Chinese	c.4898C>A, p.(Ser1633Tyr)	BR	na	na	na	[39]
			c.9260A>G, p.(Lys3087Arg)					
11228	M	Italian	c.4922C>G, p.(Ser1641*)	Bronchitis, rhinitis	SI	-	KS	[23]
			c.9304G>A, p.(Gly3102Ser)					
#5707	M	Chinese	c.5473dup, p.(Gln1825Profs*23)	na	HTX	+	CD (I+R)	[35]
			c.8275T>C, p.(Phe2759Leu)					
			c.13183C>T, p.(Arg4395*)					
#3	F	English	c.5506C>T, p.(Arg1836*)	Recurrent chest infection	SI	-	KS (Static)	[42]
			c.5636T>A, p.(Leu1879*)					
PCD565	M	Caucasian	c.5778+1G>A	NRD, BR, sinusitis, OM	SI	-	KS (H)	[22]
			c.13061T>A, p.(Leu4354His)					
PCD812	M	Caucasian	c.5815G>A, p.(Gly1939Arg)	NRD, sinusitis, OM	SI	-	KS	[22]
			c.13373C>T, p.(Pro4458Leu)					
PCD157	F	Caucasian	c.6244C>T, p.(Arg2082*)	NRD, BR, sinusitis, OM	SI	-	KS (H)	[22]
			c.11929G>T, p.(Glu3977*)					
#5130	F	Chinese	c.6785T>C, p.(Ile2262Thr)	na	HTX	+	CD (R+D)	[35]
			c.11398G>C, p.(Asp3800His)					
B178	na	Chinese	c.6905A>C, p.(His2302Pro)	BR	na	na	na	[39]
			c.13112C>T, p.(Pro4371Leu)					
B073	na	Chinese	c.7292G>T, p.(Ser2431Ile)	BR	na	na	na	[39]
			c.9017C>T, p.(Thr3006Met)					
P17	M	Chinese	c.7292G>T, p.(Ser2431Ile)	NRD, rhinosinusitis, OM	SI	-	KS (R)	[37]
P30	M		c.7364A>C, p.(Asp2455Ala)	NRD	SS	+	PCD (R)	
			c.9017C>T, p.(Thr3006Met)					
			c.13373C>T, p.(Pro4458Leu)					
#730	na	Caucasian	c.7772C>T, p.(Pro2591Leu)	NRD, rhinitis, sinusitis, OM	SS	-	PCD (erratic)	[34]
			c.8698C>T, p.(Arg2900*)					
OP98-II:1	M	Caucasian	c.7914G>C, p.Trp2604*	BR, sinusitis, OM	SI	-	KS (H)	[22]
OP98-II:2	M		c.13330_13333dup, p.(Ile4445Asnfs*4)		SS		PCD (H)	
C	M	Italian	c.8114A>G, p.(His2705Arg)	NRD, recurrent pneumonia, sinusitis, otitis	SI	+	KS (I+H)	[38]
			c.10264G>A, p.(Gly3422Arg)					
B167	na	Chinese	c.9260A>G, p.(Lys3087Arg)	BR	na	na	na	[39]
			c.11647C>T, p.(Leu3883Phe)					
B163	na	Chinese	c.9260A>G, p.(Lys3087Arg)	BR	na	na	na	[39]
			c.13175C>T, p.(Thr4392Met)					
P23	F	Chinese	c.9539T>A, p.(Leu3180*)	Rhinosinusitis, OM	SI	-	KS (R)	[37]
			c.9706C>T, p.(Arg3236*)					
#5045	M	Chinese	c.10379C>A, p.(Thr3460Lys)	na	HTX	+	CD (R+D)	[35]
			c.13273G>A, p.(Gly4425Ser)					
PCD1126	F	Asian	c.12064G>C, p.(Ala4022Pro)	BR, sinusitis	SS	-	PCD (H)	[22]
			c.13500_13504dup, p.(Thr4502Argfs*15)					
II-2	M	German	c.12363C>G, p.Tyr4121*	Recurrent pneumonia, bronchitis, sinusitis, BR (II-4, 6, 11), OM (II-3, 4, 9)	SI	-	KS	[27]
II-3, 4	F		c.13531_*36del, p.Ala4511_Ala4516delinsGln		SS		PCD	
II-6, 9, 11	M				SS		PCD	
OP235-II:1	F	Caucasian	c.12697C>T, p.(Gln4233*)	BR, sinusitis, OM	SS	-	PCD (H)	[22]
OP235-II:2	F		c.12980T>C, p.(Leu4327Ser)	NRD, BR, sinusitis, OM	SI		KS (H)	
PCD918	F	Asian	c.13065_13067del, p.(Leu4356del)	BR, NRD, sinusitis, OM	SS	-	PCD	[22]
PCD919	M		c.13075C>T, p.(Arg4359*)		HTX		KS (H)	

^#^ Description of the sequence variants is recalibrated in accordance with the Human Genome Variation Society nomenclature for variants (http://varnomen.hgvs.org/) using the reference sequence (NM_001277115.1).

BR, bronchiectasis; CD, ciliary dysfunction; CHD, congenital heart disease; D, discordance; F, female; H, hyperkinetic; HTX, heterotaxy; I, immotile; KS, Kartagener syndrome; M, male; N, nearly normal; na, not available; NRD, neonatal respiratory distress; OM, otitis media; PCD, primary ciliary dyskinesia; R, restricted; SI, situs inversus; SS, situs solitus; +, present; -, not present.

## References

[1] Xia H, Huang X, Deng S, Xu H, Yang Y, Liu X, et al. (2021) *DNAH11* compound heterozygous variants cause heterotaxy and congenital heart disease. PLOS ONE 16(6): e0252786. doi: 10.1371/journal.pone.0252786 34133440 PMC8208527

